# Maximum entropy models capture melodic styles

**DOI:** 10.1038/s41598-017-08028-4

**Published:** 2017-08-23

**Authors:** Jason Sakellariou, Francesca Tria, Vittorio Loreto, Francois Pachet

**Affiliations:** 1 SONY CSL, Paris, France; 2 Sorbonne Universités, UPMC Univ Paris 06, UMR 7606, LIP6, F-75005 Paris, France; 3 Department of Physics, Sapienza University of Rome, Rome, Italy; 4 ISI Foundation, Turin, Italy; 5 Complexity Science Hub Vienna (CSHV), Vienna, Austria

## Abstract

We introduce a Maximum Entropy model able to capture the statistics of melodies in music. The model can be used to generate new melodies that emulate the style of a given musical corpus. Instead of using the *n*–body interactions of (*n*−1)–order Markov models, traditionally used in automatic music generation, we use a k-nearest neighbour model with pairwise interactions only. In that way, we keep the number of parameters low and avoid over-fitting problems typical of Markov models. We show that long-range musical phrases don’t need to be explicitly enforced using high-order Markov interactions, but can instead emerge from multiple, competing, pairwise interactions. We validate our Maximum Entropy model by contrasting how much the generated sequences capture the style of the original corpus without plagiarizing it. To this end we use a data-compression approach to discriminate the levels of borrowing and innovation featured by the artificial sequences. Our modelling scheme outperforms both fixed-order and variable-order Markov models. This shows that, despite being based only on pairwise interactions, our scheme opens the possibility to generate musically sensible alterations of the original phrases, providing a way to generate innovation.

## Introduction

Many complex systems exhibit a highly non-trivial structure that is difficult to capture with simple models. Several biological systems form networks of interacting components (neurons, proteins, genes, whole organisms) whose collective behavior is characterized by a complex mosaic of correlations among the different components. Arguably, the ultimate biological origin of purely intellectual constructs such as language or music, should allow us to look at them from a similar point of view, i.e., as complex networks of interacting components. In both cases, one would suspect that essential features of their complexity arise from high-order combinatorial interactions. However, a number of works in recent years have shown that models based on *pairwise* interactions alone capture most of the correlation structure of some biological systems^1–6^ and even English words^7^. In this paper we extend this idea to the field of music.

One of the most popular strategies for algorithmic music composition is that of Markov chains (see for example^8^). In this setting music is seen as a sequence of symbols (these can be notes, chords, etc.) and is generated probabilistically by assigning conditional probabilities on those symbols given the preceding ones. In order to imitate the style of an existing musical corpus one can *learn* these probabilities by counting the number of occurrences of substrings of symbols, or *k*-grams, in that particular corpus. In order to capture the long-range structure of musical phrases, high-order Markov models must be used, i.e., probabilities are conditioned on (*k*−1)-grams for some large *k*. Such an approach can lead to serious over-fitting issues: the number of actually represented *k*-grams in a musical corpus is usually orders of magnitude smaller than their total potential number, which is exponential in *k*. Typical musical corpora contain a few hundred notes when the total number of different pitches is a few tens. When this is the case, probabilities for patterns longer than bi-grams (i.e., pairs of symbols) is estimated with very poor accuracy. For example, J.S. Bach’s first violin Partita contains 1910 notes when the size of the alphabet, i.e., the number of distinct notes used, is 33. In that case the number of bi-grams in the corpus and the total number of possible bi-grams are comparable, and so the estimation of bi-gram probabilities should be fairly accurate. This is, however, not true for *k*-grams with *k* greater than 2. Although this is just a particular example, pieces with a number of notes greater than quadratic to the alphabet size would be unnaturally long and are seldom found in music.

On the other hand, music is governed by a very rich and non-trivial set of rules, which may seem highly arbitrary and combinatorial. For instance in western tonal music, certain triplets of notes (such as C, E and G) are considered valid chords whereas the vast majority of three-notes combinations are rarely, if at all, used. Moreover, hardly any of these rules seems to have a fundamental character as they vary considerably across different cultures and epochs. At first sight it may seem impossible to capture the rich structure of a musical piece by a model that only takes into account pairwise information. Work in biological systems, however, has suggested that this need not be true^3^. In this paper, we show that, for musical data, enforcing pairwise consistency across different time-distances restricts the space of solutions enough for higher-order patterns to emerge. That way, we capture long range musical patterns while avoiding the over-fitting issues of high-order models. This approach cannot be implemented as an extension of Markov models, and a different framework is needed. This framework is provided by the *Maximum Entropy principle* ^9^. Maximum entropy models consistent with pairwise correlations are variations of the Ising or Potts models of statistical mechanics (see for instance^10^), which have a long and rich history as theoretical models for statistical order and phase transitions. These models belong to the large family of *Probabilistic Graphical Models*, which offer a very general framework for modeling statistical dependencies. We show here that our model can be used for generating sequences that mimic some aspects of the musical style of a given corpus.

## Results

### The Model

Music has many dimensions (melody, harmony, rhythm, form, sound, etc) which renders realistic models extremely complicated. In this paper we focus on monophonic pitch sequences, for simplicity. A pitch sequence is a sequence of integer variables {*s*_1_, …, *s*_*N*_} encoding note pitches ordered as they appear in a real melody but disregards other information about duration, onset, velocity etc. The variables take values from some finite alphabet *s*_*i*_ ∈ {1, …, *q*} which are indices of types of musical pitches. In our setting we are given an initial pitch sequence, called the *corpus* throughout the paper, of which we want to learn the style.

**Figure 1:**
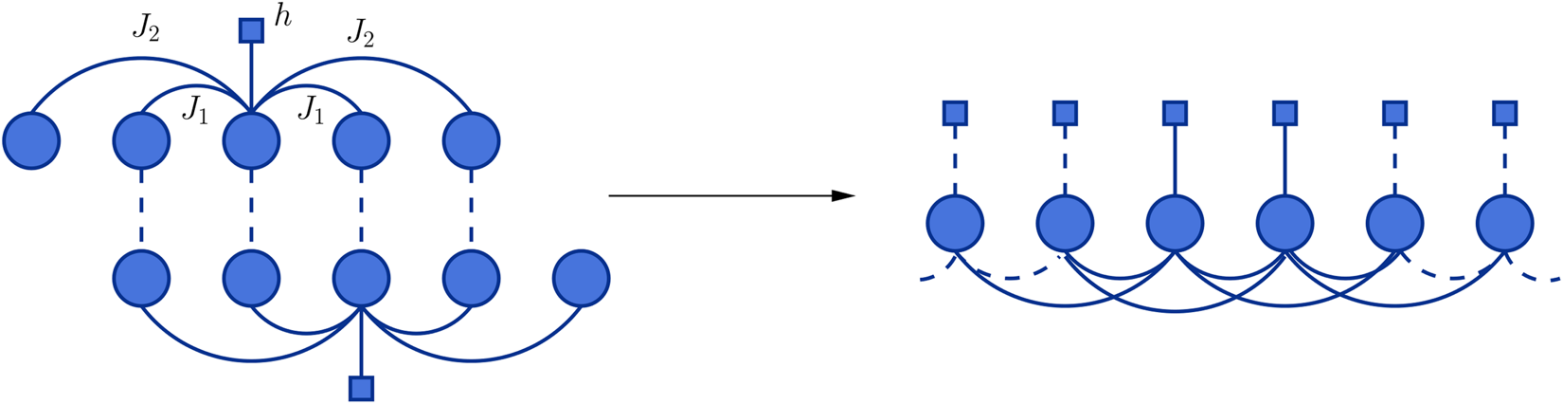
The Graph Representation. Section of a graph representing the factorization of the distribution (1) for *K*_max_ = 2. The topology of the graph reflects the way variables interact in the Hamiltonian. Interaction potentials (edges in the graph) and local fields (square nodes) are connected to variables (circle nodes) according to (1). A model is built by taking the union of smaller modules shifted by one variable, avoiding duplicate edges. Each module models the way each note depends on its local context (refer to the method section).

We will use here the The *Maximum Entropy* principle^9^, looking for the distribution *P* that maximizes the entropy $S=-{\sum }_{\{{s}_{i}\}}P({s}_{1},\ldots ,{s}_{N})\,\mathrm{log}\,P({s}_{1},\ldots ,{s}_{N})$, and reproduces the corpus frequencies of single notes and of pairs of notes at distance *k*, with *k* = 1, …, *K*_max_ (see Methods Section for details). Using Lagrange multipliers to solve the above constraint optimization problem we obtain the following Boltzmann-Gibbs distribution:

1 $$\begin{array}{ccc}P({s}_{1},\ldots ,{s}_{N}) & = & \frac{1}{Z}\,\exp \,({\sum }_{i=1}^{N}h({s}_{i})+{\sum }_{k=1}^{{K}_{max}}\sum _{{\scriptstyle \begin{array}{c}i,j\\ j-i=k\end{array}}}\,{J}_{k}({s}_{i},{s}_{j})),\\ Z & \equiv  & {\sum }_{{s}_{1}}{\sum }_{{s}_{2}}\cdots {\sum }_{{s}_{N}}\,\exp \,({\sum }_{i=1}^{N}h({s}_{i})+{\sum }_{k=1}^{{K}_{max}}\sum _{{\scriptstyle \begin{array}{c}i,j\\ j-i=k\end{array}}}\,{J}_{k}({s}_{i},{s}_{j}))\end{array}$$

where the partition function *Z* guaranties that the distribution is normalized. We will refer to the *q*-values function *h*(*s*_*i*_) as the local field, acting on each pitch of the sequence, and to the *q* ^2^-values functions *J*_*k*_(*s*_*i*_,*s*_*j*_)'s as the interaction potentials. Adopting a statistical physics point of view, these quantities can be thought as external fields acting on the variables on one hand and interactions between variables on the other hand. The Hamiltonian then gives the energy of the system by summing the contribution of all the above terms. According to distribution (1), sequences with low energy have larger probability. Therefore, the effect of the above potentials is to bias the probabilities of different sequences of notes. It is important to note that we are interested in the statistics of notes and pairs of notes independently of their exact position in the sequence. The single-note marginals should be all equal and the pair marginals should depend only on the distance between notes (refer again to the methods section). Actually, in music, position matters as the choice of notes depends strongly on a particular context. Here however we chose to focus on a *translation-invariant* (see Methods Section for details) model for simplicity, i.e., one where single and double point statistics would look the same on every neighbourhood of size of order *O*(*K*_max_). This leads to a model that is constructed by repeating a basic module which models note relations locally (see Fig. 1 and method section for more details).

In the methods section we present a method for choosing the values of these potentials in such a way as to make note frequencies of the model consistent with the ones found in a musical corpus.

Once the potentials have been found one can generate new pitch sequences by sampling from distribution (1). This can be simply done by the Metropolis Algorithm^11^. More precisely, we use the following implementation of Gibbs Sampling.
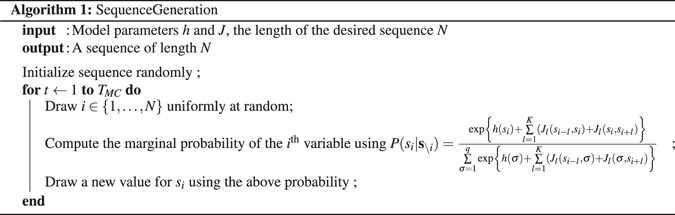


Here *T*_MC_ is the number of Monte Carlo steps. In practice we found that musically pleasing results can be obtained with a value of the order of *T*_MC_ = 10*N*. Note that in the computation of the conditional probability only the interactions with the *K*_max_ first neighbours appear.

Musical style imitation is a difficult concept to grasp and formalize. However, most musicians would agree that it involves two things: creatively rearranging existing material from the musical corpus one wants to imitate and developing the existing ideas into new ones that resemble the original material. We shall call these two activities *imitation* and *innovation*. Concerning imitation, we don’t look for an arbitrary reshuffling of substrings of notes, or *melodic patterns* as we will call them. In the new sequence, these patterns must follow “naturally” one another just as in the corpus. Concerning innovation, the new material cannot be random. One could argue that it should be statistically consistent with the corpus, by emphasizing the same notes and note pairs for example. A model that aims at imitating a given musical style should therefore be able to create music using existing melodic patterns and invented new ones in a way consistent with the corpus. We claim that our model fulfils the above criteria.

### Pairwise Correlations

**Figure 2:**
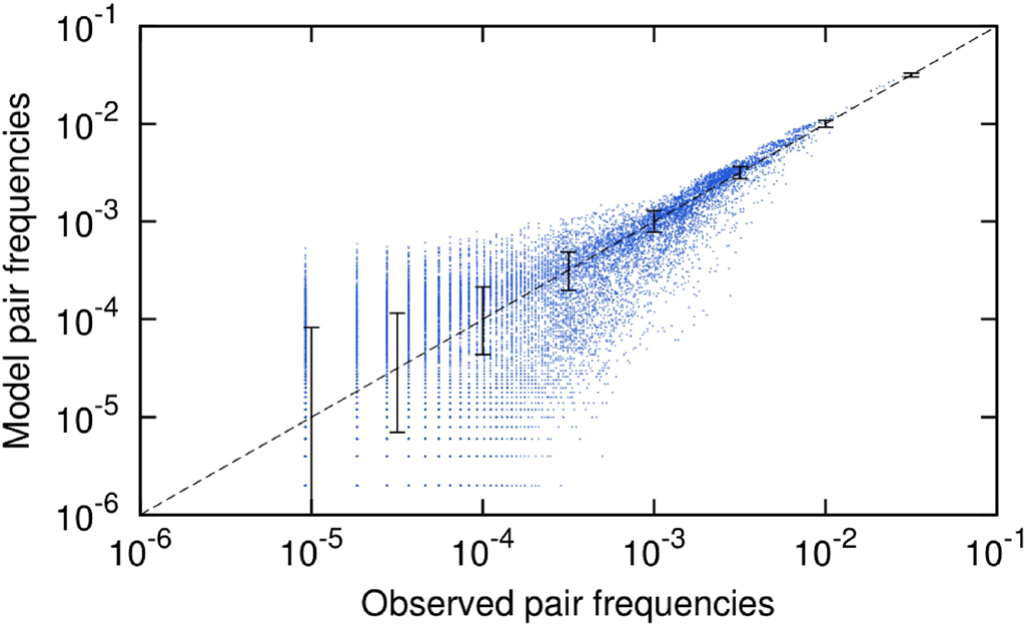
Model VS Corpus pair frequencies. The Corpus ones are from the corpus Weimar Jazz Database (that contains detailed transcriptions of famous jazz improvisations. As of march 2015 the database contains 257 songs)^12^ (see Section S3 in the SI for additional information). The model frequencies come from a *N* = 200000 sequence generated by a *K*_max_ = 10 model trained on the above corpus. Since the corpus is finite we added 99% confidence intervals for the estimated corpus frequencies. Their values were computed using the Wilson Score interval which is well adapted for values very close to 0.

We first look at what the model should do by construction: reproduce the correct single and double note frequencies. Figure 2 shows a scatter plot for pair frequencies of the corpus versus the ones generated by our model. Single note frequencies yield very similar scatter plots, just with much fewer points hence we decided not to show them. For this particular example we used as a corpus the content of the Weimar Jazz Database^12^ consisting of 257 transcriptions of famous Jazz improvisations. For more information about this corpus, as well as other corpora used in the experiments throughout this paper, we refer the reader to Section S3 of the SI. There is very good agreement for the more frequent pairs and, as expected, small probabilities are reproduced less accurately. There is a fraction of note pairs that are under-represented in generated sequences. It seems to be difficult for the basic Monte Carlo algorithm to access them. However, the great majority of note-pair probabilities are very well aligned with the corpus, as shown in Fig. 2.

**Figure 3:**
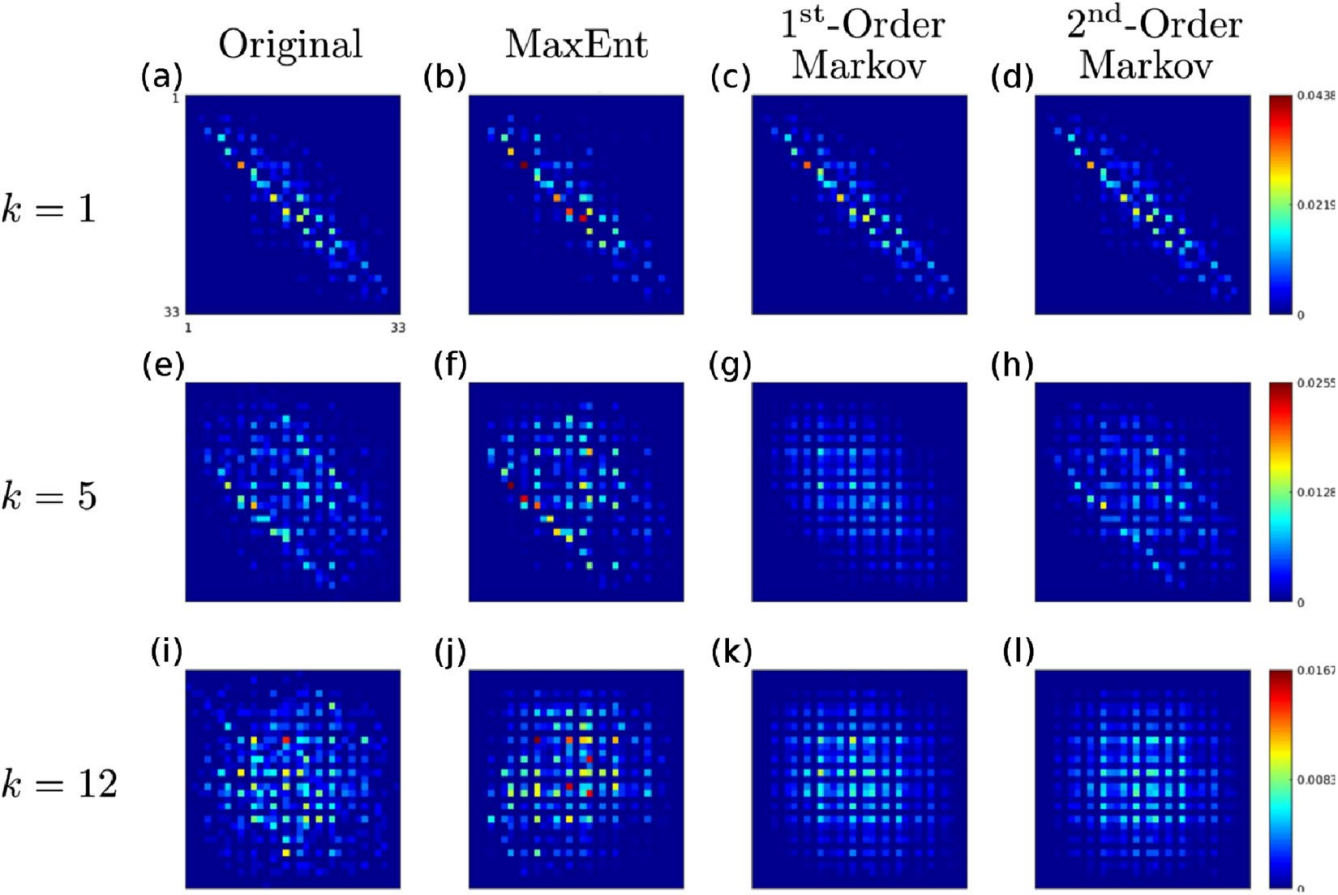
Matrices of pair frequencies. Color-maps representing matrices obtained by counting pair frequencies (see eq. (3)). (**a**–**d**) Pair frequences at distance *k* = 1 for the followig models: the original sequence by J.S. Bach (see Section S3 of the SI), our Maximum Entropy model with *K*_max_ = 10, a first-order and a second-order Markov model. (**e–h**) the same for *k* = 5. (**i–l**) the same for *k* = 12.

To better appreciate what the model does, it is informative to look at pair frequencies for different distances separately. Figure 3 shows color-maps of matrices given by eq. (3) for *k* = 1, *k* = 5 and *k* = 12 for four cases: the original sequence, here Partita No. 1 in B minor BWV 1002 by Johann Sebastian Bach part II double (see Section S3 of the SI), our maximum-entropy model (with *K*_max_ = 10), a first-order Markov model and a second-order Markov model. We contrasted the results of our model with the results of a first and second order Markov models since the sample complexity of our model is in between the sample complexity of the latter two. Our model is in fact bounded by an *O*(*K*_max_ *q* ^2^) sample complexity, even though the true sample complexity required can be safely considered an *O*(*q* ^2^) when *K*_max_ ≪ *N* since pair-frequencies at every distance can be estimated independently of each other. A first-order Markov model has quadratic in *q* complexity, and a second-order Markov model has *O*(*q* ^3^) sample complexity, i.e. comparable to *O*(*K*_max_ *q* ^2^) for *K*_max_ comparable to *q*. Visual inspection of Fig. 3 reveals that the max-entropy model not only well reproduces the pair frequencies for distances smaller than *K*_max_ (*k* = 1 and *k* = 5), but is also able to reproduce pair frequencies at greater distances (*k* = 12 in the figure). The Markov models both reproduce perfectly the correlations at *k* = 1. The first-order one, however fails completely at the greater distances. The second-order model works well at *k* = 5 but fails at larger distance. Higher-order Markov models would reproduce correlations at greater distances but at the cost of trivially copying the original sequence, as we will discuss in the following.

### Higher-order Patterns

**Figure 4:**
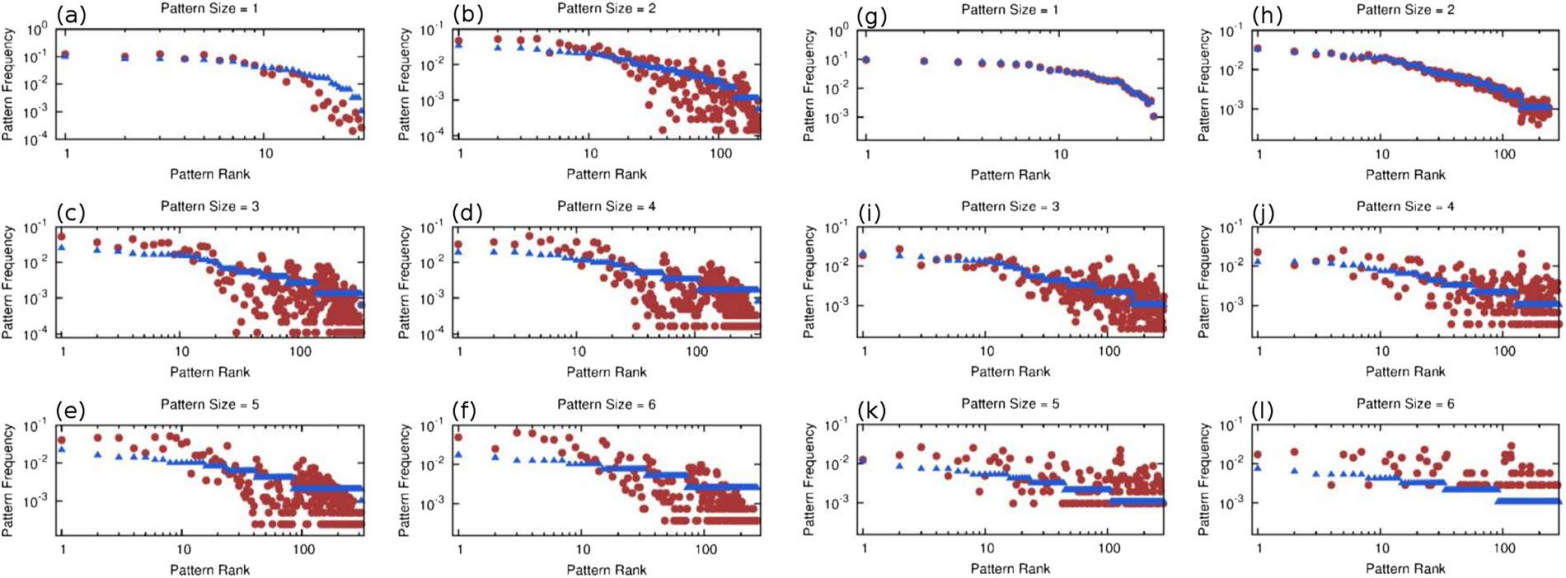
Frequency-rank plots for pattern frequencies. In blue, all patterns appearing in the corpus of sizes 1 to 6 are ranked according to their frequency. Here the corpus is the Bach Partita used previously (see Section S3 of the SI). Then, the same patterns are located in a *N* = 15000 sequence generated from the Maximum Entropy model (**a–f**) and a first-order Markov model (**g**–**l**). In red we plot their frequencies in the generated sequence but using the same order as before in order to compare with the corresponding frequencies in the corpus. For the Markov model, note that there is a good agreement for small patterns and worse for large ones. Note also that for the larger patterns (especially for size = 6) much fewer corpus patterns were found in the generated sequence, i.e. there are fewer red points than in the corresponding Maximum Entropy panel.

Pairwise correlations are explicitly enforced into the model by using pairwise interaction potentials, so it is not very surprising to correctly reproduce them. However, real music contains recognizable patterns, i.e., subsequences, of size greater than two. A music generating model should capture these higher-order patterns as well. Our model succeeds in reproducing higher-order patterns by combining multiple pairwise constraints. Figure 4 shows a series of frequency-rank plots for patterns of different sizes. Precisely, a *N* = 15000 long sequence is generated from a *K*_max_ = 30 model trained on a J.S. Bach partita (see Section S3 of the SI). Then an exhaustive search returns all patterns of sizes one up to six which are also present in the corpus. Finally we compute their frequencies, in the corpus and in the generated sequence, and plot them in decreasing order with respect to the corpus probabilities. In order to have comparable results we normalize the frequencies within the set of common patterns since our model also creates new patterns which are not present in the corpus (see next Section and Section S4 of the SI for details on this feature). The plots show that our model is indeed able to capture high-order patterns and to reproduce them with fairly consistent probabilities.

### Borrowing and Innovation

We have seen above that though the model only explicitly enforces pairwise constraints, longer melodic patterns can also be generated (see Fig. 4). We now make a step further and evaluate how well the generated melodic sequences capture the style of the original corpus without plagiarizing it. Two extremes are competing: borrowing and innovation. On the one hand the patterns generated can be identically appearing in the original corpus. The length of these patterns determines how much the generated sequence is *borrowing* from (or plagiarizing) the original corpus. If these patterns were too long one would trivially recognize the style, lacking in this way of originality. On the other hand *innovation* would imply that not all melodic patterns in the generated sequences are identical to ones found in the corpus. For example, if in a particular corpus the following patterns are present *abx*, *axc* and *xbc* with *x* substituting any character except *c*, *b* and *a* respectively, then the pattern *abc* is likely to emerge although it was never part of the corpus. We call this feature innovation as it resembles the basis of all creative processes: combining features of existing ideas to form new ones. In order to quantify the interplay between borrowing and innovation we consider suitable observables through which we evaluate the goodness of the artificial sequences generated with three methods: our Maximum Entropy model, the fixed-order Markov model and the variable-order Markov model.

We have already discussed fixed-order Markov models. In these models *k*-grams are continued according to conditional probabilities estimated from the corpus. In this case it is very difficult for instance to control the Longest Common Substring between the artificial sequence and the corpus. In Fig. S2 of the Supporting Information it can be seen that the Longest Common Substring (LCS) for fixed-order Markov grows very fast with *K*_max_, here the order of the model, leading to total plagiarism. *Variable-order Markov models* (VO) were invented^13, 14^ to circumvent this problem. Like in fixed-order Markov models each note is drawn from a distribution conditioned in the preceding *k*-gram, but this time the size *k* can vary at each step according to some criterion. A simple implementation that resolves the plagiarism problem is to use at each step of the generation the largest *k* < *K*_max_ that leads to more than, say, 3 different continuations, where *K*_max_ here is a maximal order chosen by the user. That makes plagiarism exponentially unlikely. This version of the variable-order Markov model has been successfully used in ref. 15. In these models the LCS quickly saturates to a particular value. Beyond this, changing *K*_max_ doesn’t have any effect since a much smaller *k* is always selected. As for our Maximum Entropy Model, Fig. S2 of the SI shows that the Longest Common Substring grows roughly linearly with *K*_max_.

In order to quantify the ability of the different models to capture the style of a corpus, we look at the similarity between any artificially generated sequence and the corresponding training corpus. First, in order to avoid detecting similarities/dissimilarities due to two sequences being in the same/different tonalities, we switched to a different point of view. In equal-temperament music, one can create different versions of the same melody by transposing it to a different tone. We want to treat these transposed versions as being equivalent. So, instead of encoding absolute pitch for each note, we code intervals between successive notes. In other words, we only consider the difference between successive terms in every sequence of pitches. The new sequence contains the same information as the old one, except for an additive constant which contains tonality information. Then we need a method capable of capturing some sort of similarity between these sequences of intervals.

Given two sequences ${\mathscr{A}}$ and ${\mathcal B}$ we adopt the notion of cross-complexity as a measure of the remoteness between them, the cross-complexity being the algorithmic version of cross-entropy^16, 17^. We define the cross-complexity (though here we shall refer to it as cross-entropy) of a sequence ${\mathcal B}$ with respect to another sequence ${\mathscr{A}}$ as the amount of bits needed to specify ${\mathcal B}$ once ${\mathscr{A}}$ is known. We follow a refined version of the data-compression approach introduced in refs 18, 19, that was shown to be successful in authorship attribution and corpora classification^19^. We use in particular the LZ77 compressor^20^ and we scan the ${\mathcal B}$ sequence looking for existing matching sub-sequences only in ${\mathscr{A}}$ and we code each matching as in the usual LZ77 algorithm. In this way we estimate the cross-entropy of each artificially generated sequence with respect to the corresponding sequence of the original corpus. Details about the usage of data compression techniques to estimate the cross-entropy between two sequences are reported in the Supporting Information.

The cross-entropy described so far helps us in quantifying the similarity of the artificially generated sequences with the original corpus. The smaller the value of the cross-entropy the larger is the similarity. Now a small-value of the cross-entropy may be due to a genuine *stylistic* similarity between the two sequences. In this case the artificial sequence looks like the original corpus without plagiarizing it, i.e., without borrowing large subsequences of the corpus itself. On the other hand, a small cross-entropy may be due to the presence of large chunks of the original corpus in the artificial sequence. To discriminate between these two cases we look at another observable, namely the *Average Common Substring* (ACS) between the artificial sequence and the corpus. The ACS is also computed using the data-compression technique described above. Given two sequences ${\mathscr{A}}$ and ${\mathcal B}$ and all the substrings found by the LZ77 algorithm while parsing ${\mathcal B}$ for matching in ${\mathscr{A}}$, the ACS is defined as the average length of all the matches found. We compute the ACS of each artificially generated sequence with respect to the corresponding sequence of the original corpus. Small ACS implies an important degree of innovation in the artificial sequence, while a large value of ACS implies a high degree of borrowing.

**Figure 5:**
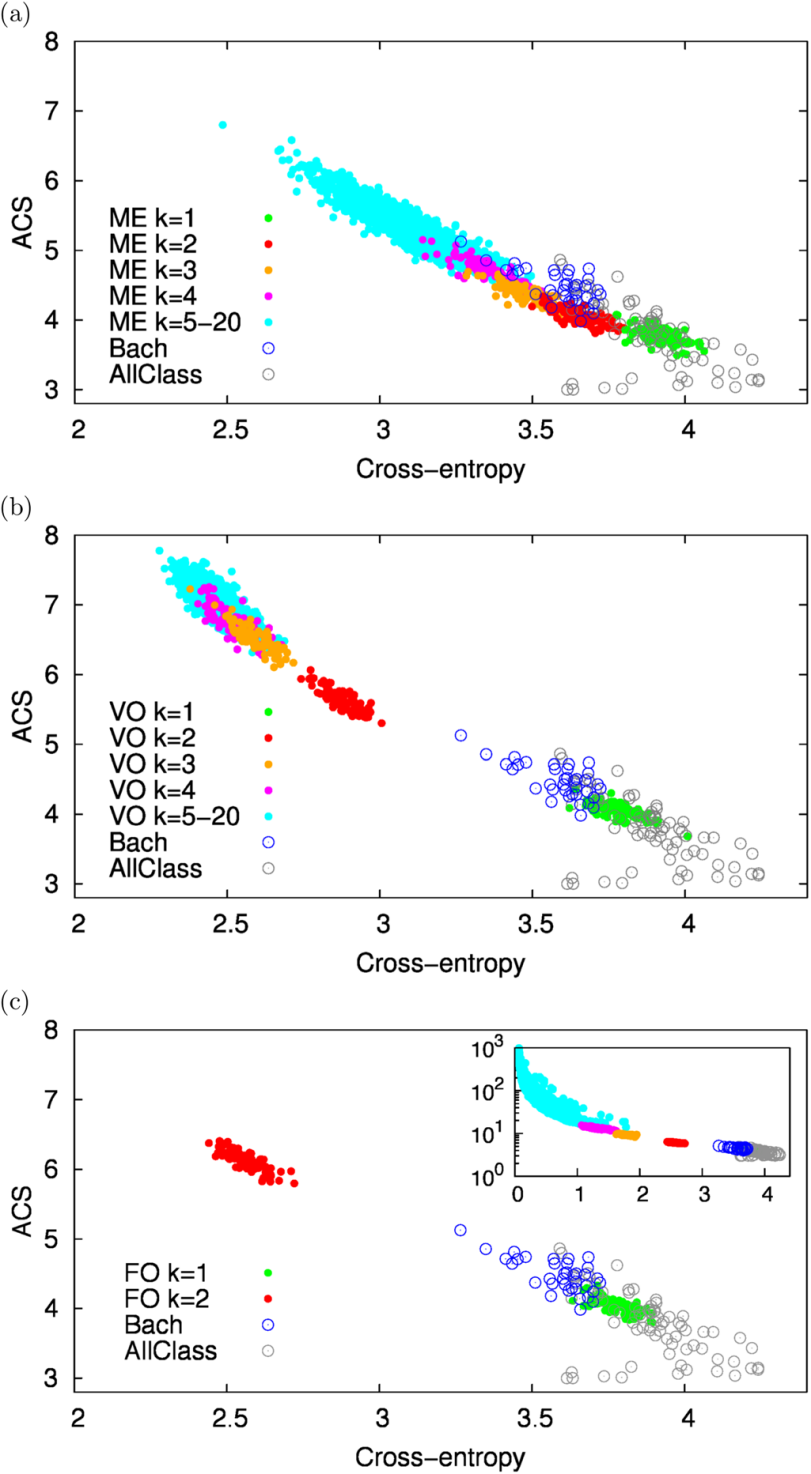
Borrowing vs. similarity. This figure reports the Average Common Substring (ACS) vs. the values of the cross-entropies for all the artificial sequences generated with the Maximum Entropy (ME) model (**a**), the variable-order (VO) Markov model (**b**) and the fixed-order (FO) Markov model **(c)**. Everything is computed with respect to the sequence of J.S. Bach’s first violin Partita (see Section S3 of the SI). Filled circles correspond to the artificial sequences. Colors code for the values of *K*_max_ in each different model. In addition in each panel the empty circles reports the same quantities for other original sequences of Bach (represented with blue circles) and other classical authors - Beethoven, Schumann, Chopin, Liszt and Albeniz - (AllClass represented with grey circles). Note that the main panel for FO is truncated at values of ACS equal to 8, while the complete plot is shown in the inset.

Overall, while the cross-entropy informs us about how statistically similar is the artificially generated sequence to the original corpus, the ACS tells us about the degree of borrowing from the original corpus. Figure 5 illustrates the results of this analysis performed using J.S. Bach’s first violin Partita in B minor as the original corpus. It is clear that the Maximum Entropy model is the only one able to capture values of similarity and level of borrowing comparable to those of other corpora from Bach (blue circles). Both variable-order and fixed order Markov models either feature a low level of borrowing but large dissimilarities (green filled circles for *k* = 1) or high similarity with the original corpus but large values of ACS, i.e., a high level of borrowing. From this perspective the Maximum Entropy model features an optimal balance between similarity with the original corpus with a level of borrowing comparable to that found between different original pieces of Bach. Similar results for other original corpora (from Beethoven, Schumann, Chopin, Liszt) are reported in the Supporting Information. A similar analysis is also reported in the SI, where LCS is considered instead of ACS, bringing to similar results. Finally, in order to let the reader to evaluate “musically” the artificial pieces generated by our Maximum Entropy model, we provide, as audio Supporting Information, a series of wav files including original pieces (both classical and jazz) and the corresponding artificially generated ones (see for details Section S7 of the textual Supporting Information).

## Discussion

We presented a Maximum Entropy model that captures pairwise correlations between notes in a musical sequence, at various distances. The model is used to generate original sequences that mimic a given musical style. The particular topology of this model (see Fig. 1) leads to the emergence of high-order patterns, despite the pairwise nature of the information used, which in turn has the benefits of a quadratic, in the alphabet size, sample complexity. Moreover, the absence of high-order constraints and their substitution by multiple pairwise constraints, gives our model more freedom to create new melodic material that imitates the musical style of a given corpus.

One key questions arising when proposing a specific algorithm to generate artificial musical sequences with the *style* of a given corpus is how to validate the results, i.e., to provide a quantitative account of how much the sequences generated by the model are similar to the original corpus without plagiarizing it. To this end we considered two specific observables to quantify the levels of borrowing and innovation in the generated sequences. Based on data-compression techniques, these two observables allow to claim that Maximum Entropy models, like the one proposed here, outperform both fixed-order and variable-order Markov models in providing musically sensible alternative realizations of the style of a given corpus.

Graphical models like the one proposed here are ideal for implementing user control over the generated sequence. Any desired note can be constrained by the user in advance and these constraints will be propagated to the rest of the notes during the Monte Carlo procedure, biasing the generation towards music that is consistent with the constraints. Finally graphical models offer a general framework for modeling statistical dependencies. Work is in progress to extend this modeling scheme in order to account for other aspects of music, such as rhythm, polyphony and expressivity. The general idea is the same: additional information (e.g., note durations) can be captured by additional variables coupled with *pairwise* interactions. In that way, one can keep the quadratic sample complexity, while avoiding over-fitting, and create models able to make musically sensible generalizations of the ideas found in the corpus.

## Methods

### Model details

We are interested in reproducing the corpus frequencies of single notes and of pairs of notes at distance *k*,

2 $$f(\sigma )\equiv \frac{1}{N}\sum _{i\mathrm{=1}}^{N}\delta (\sigma ,{s}_{i})$$

3 $${f}_{k}(\sigma ,\sigma {\rm{^{\prime} }})\equiv \frac{1}{N-k}\sum _{{\scriptstyle \begin{array}{c}i,j\\ |j-i|=k\end{array}}}\delta (\sigma ,{s}_{i})\delta (\sigma {\rm{^{\prime} }},{s}_{j})$$

with *k* = 1, …, *K*_max_. In the above formulas *δ*(⋅, ⋅) is the Kronecker delta symbol. The sums run over the whole corpus.

It is crucial to note that the above quantities have no dependence on the position within the sequence, i.e., they do not depend on the indices *i* and *j*. The first quantity in eq. (2) represents the frequency of notes, regardless of the position at which they appear. The quantity in eq. (3) represents the frequency of co-occurrence of pairs of notes, again regardless of position, depending however on the distance between the variables.

We look for the distribution (or probabilistic model) *P* that maximizes the entropy $S=$ $-{\sum }_{\{{s}_{i}\}}\,P({s}_{1},\ldots ,{s}_{N})\,\mathrm{log}\,P({s}_{1},\ldots ,{s}_{N})$ (Maximum Entropy principle) and that satisfies:

4 $$f(\sigma )={P}_{i}\,({s}_{i}=\sigma )=\sum _{\{{s}_{k}|k\ne i\}}P({s}_{1},\ldots ,{s}_{N}),\quad {\rm{\forall }}i$$

and

$${f}_{k}(\sigma ,\sigma {\rm{^{\prime} }})={P}_{ij,k}({s}_{i}=\sigma ,{s}_{j}=\sigma {\rm{^{\prime} }})=\sum _{\{{s}_{l}|l\ne i,j\}}P(\ldots ,{s}_{i}=\sigma ,\ldots ,{s}_{j}=\sigma {\rm{^{\prime} }},\ldots )\quad {\rm{\forall }}i,j:|j-i|=k$$

where in the right hand side we have the marginals of the model’s distribution.

Note that one-note marginals are the same for every note *i* and two-notes marginals are the same as long as the notes *i*, *j* are the same distance apart *k*. The model can have any desired length, i.e., any number of notes. However, the interactions between variables extend to a maximum length given by *K*_max_ which is usually much smaller than the total length of the model. Moreover, interactions for same-distance variables repeat themselves along the graphical model, as described earlier. The interaction graph is therefore highly regular and can be seen as constructed from some basic module. This module is composed of one variable node, its local field and all its first-neighbours. It has size 2*K*_max_ + 1 and contains a copy of all the parameters of the model, i.e., one local field *h* and two copies of each interaction potential *J*_*k*_. Each module models the way each note depends on its local context. In order to build a bigger model we take the union of two such modules shifted by one variable, avoiding duplicate edges, as shown in Fig. 1. This procedure is then repeated a number of times until the desired total number of variables *N* is reached. That creates a translation invariant model, except for regions of size *K*_max_ on the borders, which will have a negligible effect since *K*_max_ ≪ *N* (see for details Section S2 of the Supporting Information). At generation time, one can generate larger than desired sequences and discard border sections.

The above picture of a longer model built by the union of simpler modules allows us to simplify the task of choosing the values of the potential in eq. (1) by realizing that we need to infer the potentials only for one such module.

### Parameter learning

We use a *Maximum Likelihood Estimation* (MLE) of the model parameters, i.e., we want to minimize the negative log-likelihood

5 $$ {\mathcal L} (\{{J}_{k}\},h|{\bf{s}})=-\frac{1}{M}\sum _{\mu \mathrm{=1}}^{M}\,\mathrm{log}\,P({{\bf{s}}}^{(\mu )})$$

where *M* is the number of samples at our disposal and the superscipt *μ* labels the samples of the dataset. The minimization of the above function is very difficult in general due to the intractability of the partition function *Z* in (1), so we have to resort to an approximate method. Among many possible approximate methods for solving the above problem we chose the *pseudo-likelihood* maximization approach, introduced in^21^, for reasons that will become clear below. The original paper treats models with binary variables such as the Ising model. A generalization to multi-valued variables, such as in our case, can be found in ref. 22 where the authors use a very similar model to ours to infer interactions between amino-acids in protein-protein interactions.

**Figure 6:**
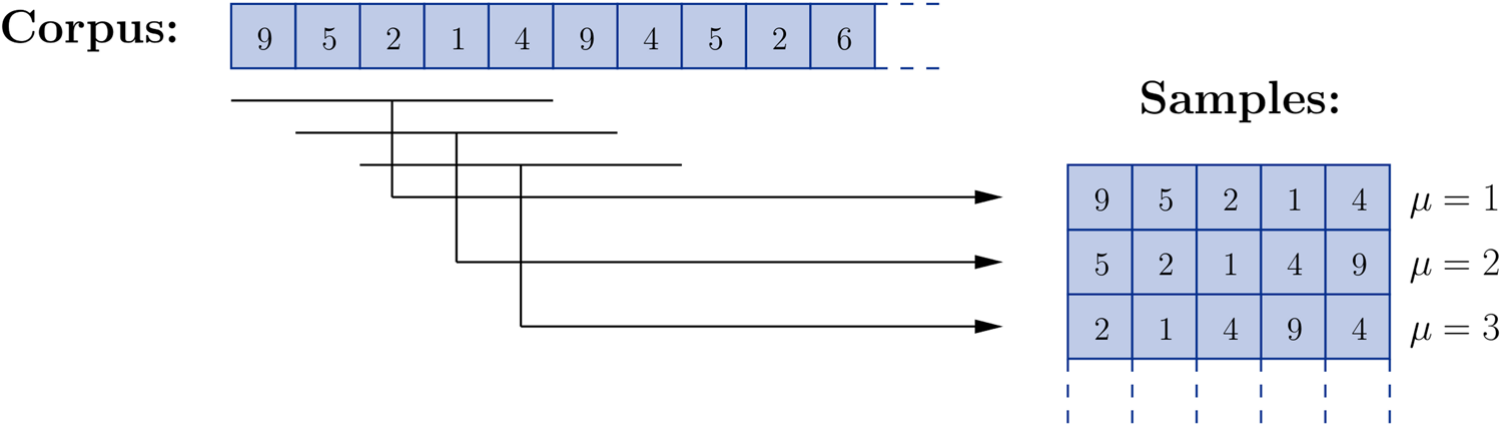
Training data. The corpus, a sequence of type indices, is segmented by overlapping substrings of size 2*K*_max_ + 1 (here *K*_max_ = 2). These samples provide the information needed to train the basic module of our model, which describes the way a variable depends on its local context, i.e., on *K*_max_ variables to its left and to its right.

In the pseudo-likelihood approximation one replaces, in the minimization task, the full probability of the model with the conditional probabilities of the variables given their neighbors and, in that way, replaces the original problem with a set of logistic regression problems. Each neighborhood is inferred independently and then the information is combined to get the full interaction graph. The authors in ref. 21 have shown that this approximation works well under certain conditions. In physics terminology, as long as the variables are not interacting too strongly, treating the neighborhoods independently gives fairly good results. We have found empirically that the parameters inferred from musical data fall into this category since we are able to reproduce the corpus note frequencies fairly well. As we described in the previous section, the model can be decomposed in a number of identical copies of some basic module. This particular structure makes the pseudo-likelihood a particularly appropriate method since the parameters on a neighbourhood have to be inferred only once. Moreover, the above picture leads to a natural way to segment the corpus into samples: the samples used in the training phase are all the substrings of size 2*K*_max_ + 1 in the corpus, each providing the necessary information to model the way a variable depends on its local context, i.e., on *K*_max_ variables to its left and to its right, see Fig. 6. There is a harmless redundancy in the data since most note pairs are used twice when inferring the interaction potentials *J*_*k*_, except for a negligible number of pairs of order *O*(*K*_max_) at the corpus’ borders.

In our case, the conditional probability used in the pseudo-likelihood approach is written

6 $$P({s}_{0}^{(\mu )}|{{\bf{s}}}_{\backslash 0}^{(\mu )})=\frac{\exp \{h({s}_{0}^{(\mu )})+\sum _{l=1}^{K}({J}_{l}({s}_{-l}^{(\mu )},{s}_{0}^{(\mu )})+{J}_{l}({s}_{0}^{(\mu )},{s}_{l}^{(\mu )}))\}}{\sum _{\sigma =1}^{q}\exp \{h(\sigma )+\sum _{l=1}^{K}({J}_{l}({s}_{-l}^{(\mu )},\sigma )+{J}_{l}(\sigma ,{s}_{l}^{(\mu )}))\}},$$

where the index *μ* represents the *μ* ^th^ sample, ${s}_{0}^{(\mu )}$ is the central variable, ${{\bf{s}}}_{\backslash 0}^{(\mu )}$ represents the remaining variables in the neighborhood, and ${s}_{-l}^{(\mu )}$ and ${s}_{l}^{(\mu )}$ ^th^e *l* ^th^ variable to the left and to the right of the central one respectively. The advantage of the above method lies in the tractability of the normalization in (6) as opposed to the one in (1). The log-pseudo-likelihood function is

7 $${ {\mathcal L} }_{{\rm{pseudo}}}(\{{J}_{k}\},h|{\rm{S}})=-\frac{1}{M}\sum _{\mu \mathrm{=1}}^{M}\,\mathrm{log}\,P({s}_{0}^{(\mu )}|{{\bf{s}}}_{\backslash 0}^{(\mu )}),$$

where the sum rums over all samples, i.e., all substrings of length 2*K*_max_ + 1 of the corpus. Minimizing the above function yields the potentials of the whole model since they are repeated on every neighborhood. In addition, one usually adds a regularization term to avoid over-fitting issues (see Section S1 of the SI). For the details concerning the optimization procedure see Section S2 of the SI.

## Electronic supplementary material

Audio file 44

Audio file 45

Audio file 46

Audio file 47

Audio file 33

Audio file 34

Audio file 35

Audio file 36

Audio file 37

Audio file 38

Audio file 39

Audio file 40

Audio file 41

Audio file 42

Audio file 43

Audio file 29

Audio file 30

Audio file 31

Audio file 32

Audio file 1

Audio file 2

Audio file 3

Audio file 4

Audio file 5

Audio file 6

Audio file 7

Audio file 8

Audio file 9

Audio file 10

Audio file 11

Audio file 12

Audio file 13

Audio file 14

Audio file 15

Audio file 16

Audio file 17

Audio file 18

Audio file 19

Audio file 20

Audio file 21

Audio file 22

Audio file 23

Audio file 24

Audio file 25

Audio file 26

Audio file 27

Audio file 28

Audio file 48

Supporting Information
