# Supplementary material for: Maximum entropy models capture melodic styles
Source: Sci Rep. 2017 Aug 23;7:9172. doi: 10.1038/s41598-017-08028-4 (PMC5569059; doi:10.1038/s41598-017-08028-4)
Supplement: Supplementary file 49 — Supporting Information [file 41598_2017_8028_MOESM49_ESM.pdf]

Maximum entropy models capture melodic styles

Supporting Information

Jason Sakellariou[1,2], Francesca Tria[3,4], Vittorio Loreto[3,4] and Francois Pachet[1,2]

[1] SONY CSL, Paris, France

[2] Sorbonne Universités, UPMC Univ Paris 06, UMR 7606, LIP6, F-75005, Paris, France

[3] Department of Physics, Sapienza University of Rome, Rome, Italy

[4] ISI Foundation, Turin, Italy

S1 Regularization procedure

In addition to the log-likelihood defined in the main text (reported here below for convenience)

$$\mathcal{L}_{\text{pseudo}}(\{J_k\}, h|\mathsf{S}) = -\frac{1}{M}\sum_{\mu=1}^{M} \log P(s_0^{(\mu)}|\mathbf{s}_{\setminus 0}^{(\mu)}) \quad , \tag{1}$$

one usually adds a regularization term. Regularization is motivated from multiple points of view, the main of which is to avoid over-fitting. From the point of view of our model, regularization has an additional benefit. It eliminates the degeneracy in the choice of parameters in the model. The model given by

$$\mathcal{H}(s_1, \ldots, s_N) = -\sum_{i=1}^{N} h(s_i) - \sum_{k=1}^{K_{\max}} \sum_{\substack{i<j \\ |i-j|=k}} J_k(s_i, s_j) \quad . \tag{2}$$

exhibits *gauge invariance*, i.e., there are different choices of parameters J_k and h which assign the same probabilities to the same variable configurations (see for example [1]). In order to have unique and reproducible results for a given training set we wish to remove this degeneracy. The addition of a ℓ_p-norm regularizer does that by yielding the solution which minimizes the ℓ_p-norm

of the interaction matrices J_k.

Specifically, here we use ℓ_1-norm regularization, pioneered in [2], and adapted in the context of sparse model selection in [3]. The benefits of ℓ_1-norm regularization are twofold. First, it yields sparse results by forcing a substantial number of parameters to zero. This is reasonable in our case since our starting information is sparse: out of all the possible note-pairs only a fraction is actually used. Thus it wouldn't make much sense to infer a number of parameters much larger than the number of independent quantities actually observed. The second advantage of the ℓ_1-norm is that it conserves the convexity of the objective function.

Concretely, instead of the log-likelihood function in eq. (1) we use:

$$\mathcal{L}_{\text{pseudo, reg}}(\{J_k\}, h|\mathsf{S}) = -\frac{1}{M}\sum_{\mu=1}^{M} \log P(s_0^{(\mu)}|\mathbf{s}_{\backslash 0}^{(\mu)}) + \frac{\lambda}{M}\sum_{k=1}^{K_{\max}} \|J_k\|_1 \quad . \tag{3}$$

By tuning the parameter λ one can force the minimization procedure to set more or less values of the matrices J_k equal to zero. In practice, we found that values between $\lambda = 1$ and 3 yielded the smallest MSE between model and corpus pair-note probabilities.

S2 Optimization

We start by writing explicitly the negative log-likelihood. Since we use the pseudo-likelihood approach we will use the following conditional probability

$$P(s_0^{(\mu)}|\mathbf{s}_{\backslash 0}^{(\mu)}) = \frac{\exp\left\{h(s_0^{(\mu)}) + \sum_{l=1}^{K}\left(J_l(s_{-l}^{(\mu)}, s_0^{(\mu)}) + J_l(s_0^{(\mu)}, s_l^{(\mu)})\right)\right\}}{\sum_{\sigma=1}^{q}\exp\left\{h(\sigma) + \sum_{l=1}^{K}\left(J_l(s_{-l}^{(\mu)}, \sigma) + J_l(\sigma, s_l^{(\mu)})\right)\right\}} \quad , \tag{4}$$

The negative logarithm of the pseudo-likelihood together with the regularization term then reads

$$
\begin{aligned}
\mathcal{L}_{\text{pseudo, reg}}(\{J_k\}, h|\mathsf{S}) &= -\frac{1}{M}\sum_{\mu=1}^{M} \log P(s_0^{(\mu)}|\mathbf{s}_{\backslash 0}^{(\mu)}) \\
&= -\frac{1}{M}\sum_{\mu=1}^{M}\Bigg\{ h(s_0^{(\mu)}) + \sum_{k=1}^{K_{\max}} \Big(J_k(s_{-l}^{(\mu)}, s_0^{(\mu)}) + J_k(s_0^{(\mu)}, s_{+l}^{(\mu)}) \Big) \\
&\qquad\qquad - \log Z^{(\mu)} \Bigg\} + \frac{\lambda}{M}\sum_{k=1}^{K_{\max}} \|J_k\|_1 \quad ,
\end{aligned} \tag{5}
$$

where the partition function is

$$
Z^{(\mu)} = \sum_{\sigma=1}^{q} \exp\left\{ h(\sigma) + \sum_{l=1}^{K_{\max}} \Big(J_l(s_{-l}^{(\mu)}, \sigma) + J_l(\sigma, s_l^{(\mu)}) \Big) \right\} \quad . \tag{6}
$$

For the minimization of the above function we used an ℓ_1-regularized problem solver written in Matlab by Mark Schmidt [4, 5]. Specifically, we used the projected scaled sub-gradient (Gafni-Bertsekas variant). The solver makes use of the gradient of the above function, provided by the user. The gradient elements have two forms depending on whether one is differentiating with respect to a local field or an interaction potential. The two forms are respectively

$$
\begin{aligned}
\frac{\partial \mathcal{L}}{\partial h(r)} &= -\frac{1}{M}\sum_{\mu=1}^{M}\left(\delta(s_0^{(\mu)}, r) - \frac{\partial}{\partial h(r)} \log Z^{(\mu)} \right) \\
&= -\frac{1}{M}\sum_{\mu=1}^{M}\left(\delta(s_0^{(\mu)}, r) - \frac{1}{Z^{(\mu)}} \exp\left\{ h(r) + \sum_{l=1}^{K_{\max}} \Big(J_l(s_{-l}^{(\mu)}, r) + J_l(r, s_l^{(\mu)}) \Big) \right\} \right)
\end{aligned} \tag{7}
$$

and

$$
\begin{aligned}
\frac{\partial \mathcal{L}}{\partial J_k(r,r')} &= -\frac{1}{M}\sum_{\mu=1}^{M}\left(\delta(s_{-k}^{(\mu)},r)\delta(s_0^{(\mu)},r') + \delta(s_0^{(\mu)},r)\delta(s_{+k}^{(\mu)},r') - \frac{\partial}{\partial J_k(r,r')}\log Z^{(\mu)}\right) + \frac{\lambda}{M}|J_k(r,r')| \\
&= -\frac{1}{M}\sum_{\mu=1}^{M}\Bigg(\delta(s_{-k}^{(\mu)},r)\delta(s_0^{(\mu)},r') + \delta(s_0^{(\mu)},r)\delta(s_{+k}^{(\mu)},r') \\
&\qquad - \frac{1}{Z^{(\mu)}}\delta(s_{-k}^{(\mu)},r)\exp\left\{h(r') + \sum_{l=1}^{K_{\max}}\left(J_l(s_{-l}^{(\mu)},r') + J_l(r',s_l^{(\mu)})\right)\right\} \\
&\qquad - \frac{1}{Z^{(\mu)}}\delta(s_{+k}^{(\mu)},r')\exp\left\{h(r) + \sum_{l=1}^{K_{\max}}\left(J_l(s_{-l}^{(\mu)},r) + J_l(r,s_l^{(\mu)})\right)\right\}\Bigg) + \frac{\lambda}{M}|J_k(r,r')| \quad .
\end{aligned}
\tag{8}
$$

Note that the first terms in the above equation are counting the number of occurrences of some event in the samples. The first term in eq. (7) counts the number of samples where the central variable takes the value r. The first and second terms in eq. (8) count the number of samples where the pair r, r' appears at distance k and where one of the two variables is the central one. These quantities can be precomputed. The remaining terms (except the regularization) are the model predictions for these same quantities, so finding the minimum of the function in eq. (5) equates the model frequencies with the empirical ones.

Border Terms

Note that the way to segment the corpus and the way to train the model, as described in the Methods section of the main text, yield a model that is not entirely translation-invariant. Each sample contains a midle target-note, $K_{\max}$ neighbors to the left and $K_{\max}$ to the right. This means that the first and last $K_{\max}$ notes of evey piece in the corpus are never used as target-notes. They also contribute less in the pair frequencies as every other pair of notes appears twice in the dataset. We chose this scheme for its simplicity, since the border sections contributions are

negligible. Indeed, since we always work in a $K_{\max} \ll N$ regime, final results are barely affected by this choice. In support for this claim we provide Fig. S1 where single-note and pair frequencies of the WJDB corpus (see section **S3**) are plotted with and without taking into account border terms of size $K_{\max} = 10$. The agreement is obviously very good.

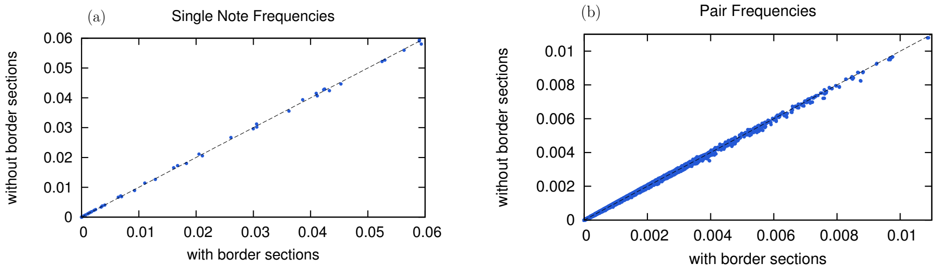

FIG. S1: **Border Sections Contribution.** The figure shows single-note frequencies **(a)** and pair frequencies **(b)** computed from the WJDB corpus (see section **S3**) with and without taking border sections of size 10 into account.

S3 Musical Corpora

We used a variety of musical corpora in our experiments. Since our model is by construction monophonic we either used monophonic music or extracted monophonic sequences from polyphonic pieces. Additionally, we ignored rhythm and discarded all information about note onsets and durrations. All corpora where taken from the following two databases:

- The **Weimar Jazz Database (WJDB)**[6] contains detailed transcriptions of famous jazz improvisations. As of March 2015 the database contains 257 songs. The majority of improvisations come from brass and wind instruments so they are monophonic by default.

- **Kunst der Fuge (KdF)**[7] is a classical music MIDI files database. It

contains thousands of classical music pieces in MIDI format. Most of the tunes are polyphonic so, in that case, we extracted separate tracks from the MIDI files when possible and chose our sequences among them. When necessary, we discarded simultaneous notes by keeping the highest one.

To be more specific, the sequences used as training corpora in this paper were exctracted from the following works.

In addition, in the figures Fig. 6, Fig. S4, Fig. S5 and Fig. S6, the borrowing and similarity features where also computed against a large number of sequences called *AllClass*, extracted from the KdF database and coming from composers Albeniz, Bach, Beethoven, Chopin, Liszt and Schumann. In these figures, as a reference for the evaluation of the models, we compute additionally the borrowing and similarity features between the training corpus and the above mentioned sequences. We discriminate between sequences from the same composer (blue non-filled circles) and sequences of all other composers (grey non-filled circles). In order to avoid different tonalities issues we used sequences of intervals (see main text, section **borrowing and innovation**).

S4 Similarity vs. Innovation

In the main text we have seen that our model is able to reproduce melodic patterns from the corpus of different sizes, although the model only explicitly enforces pairwise constraints. We have also quantified the interplay between borrowing and innovation in the artificial sequences generated starting from a given corpus. Here innovation means that not all melodic

TABLE S1: Musical pieces used as training corpora in this work

**Figure**	**Data Base**	**Work**
Fig. 3 , Fig. S1	WJDB	The whole database
Fig. 4, Fig. 5, Fig. 6 Fig. S2,Fig. S3	KdF	Violin Partita No.1 in B minor, BWV 1002 (Bach, Johann Sebastian)
Fig. S4	KdF	– 11 Bagatelles, Op.119 (Beethoven, Ludwig van)<br>– Movement I, Symphony No.8, Op.93 (Beethoven, Ludwig van)<br>– 3 Marches, Op.45 (Beethoven, Ludwig van)
Fig. S5	KdF	– Andante und Variationen, Op.46 (Schumann, Robert)<br>– Mazurka No.1, Op.56 (Chopin, Frdric)<br>– Liebesträume No.3, S.541 (Liszt, Franz)
Fig. S6	KdF	Violin Partita No.1 in B minor, BWV 1002 (Bach, Johann Sebastian)

patterns in the generated sequences are identical to ones found in the corpus. Although these new patterns might not be part of the corpus, they are far from being random. They emerge from the effort to satisfy multiple competing pairwise constraints and for that reason they are “musically” interesting. When a musician decides to substitute a few in a given phrase,

the new notes will bear relations with the remaining notes that are probably found elsewhere in the corpus. This is exactly what our model does.

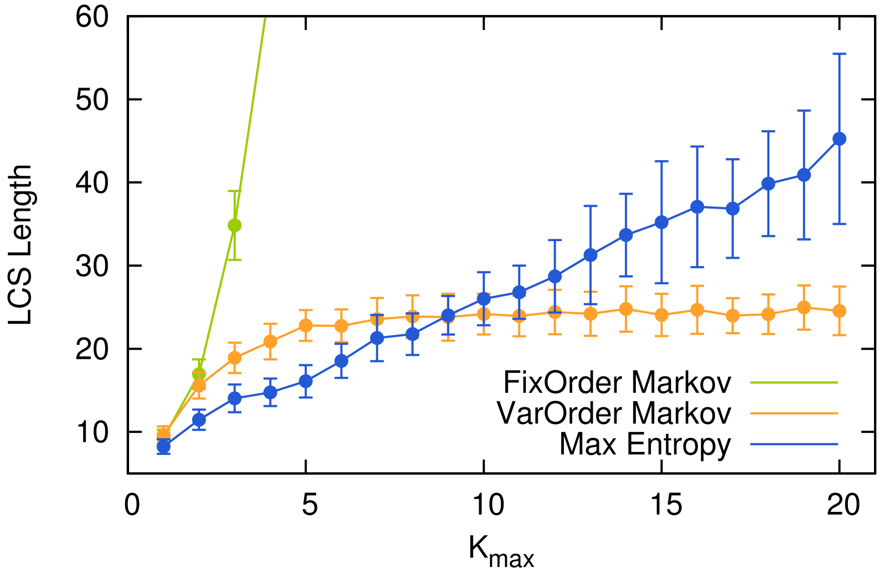

FIG. S2: **Longest Common Substrings** between the original corpus (see Section S3 for details) and the sequences (of length $N = 5000$) artificially generate with the three models considered in the main text. Results are obtained by averaging over 100 sequences for each model.

We first focus on the level of borrowing or imitation. We study in particular the behavior of the *Longest Common Substring* (LCS) between the original and generated sequences. This feature provides some insight about the balance between style imitation on one hand and plagiarism on the other. As we said earlier, style imitation consists, among other things, in re-arranging existing melodic patterns. If these patterns are too long the new sequence will give the impression of copying the corpus instead of imitating it. If on the other hand these patterns are too short, the style of the corpus will not be recognizable. It would be

then desirable to be able to control the size of the longest common substring (LCS) in the generated sequence in order to find the right balance between imitation and innovation.

Fig. S2 illustrates how the LCS for fixed-order Markov grows very fast with $K_{\max}$, here the order of the model, leading to total plagiarism. On the other hand for variable-order Markov models the LCS quickly saturates to a particular value. Beyond this point changing $K_{\max}$ does not have any effects since a much small k is always selected. In contrast, in our Maximum Entropy model the size of LCS scales linearly with $K_{\max}$. As we discussed earlier, this property is desirable since it allows to fine-tune the balance between style imitation and plagiarism.

In order to further picture the rates of borrowing and innovation of our model we did the following additional experiment. First we count the number of all distinct patterns that appear in the corpus as a function of pattern size $n_{\text{corpus}}(l)$. Then we generate sequences of various sizes and count the number of distinct patterns in them $n^{(d)}_{\text{generated}}(l)$ at a given Hamming distance d from patterns also appearing in the corpus, or less. The Hamming distance between two patterns is simply the number of positions at which the sequences have different symbols. For example, if pattern *abcd* appears in the corpus, then e.g., pattern *abxd* with any x is at distance $d = 1$ if $x \neq c$ and $d = 0$ for $x = c$. Finally we plot the fraction $n^{(d)}_{\text{generated}}(l)/n_{\text{corpus}}(l)$ for $d = 0$ and $d = 1$. When we count the number of patterns at $d = 0$ we are quantifying the degree of imitation of our model, since these patterns appear as such in the corpus. On the other hand, when we count the number of patterns at $d = 1$ we have some indication about the degree of innovation of our model. Fig. S3 reports the results. The red curves correspond to distance $d = 0$ whereas the blue curves correspond to $d = 1$. As a refference we have also counted the number of all possible patterns at distance $d = 1$

from the patterns of the corpus, which we obtained by simple enumeration, and included the corresponding curve (black line).

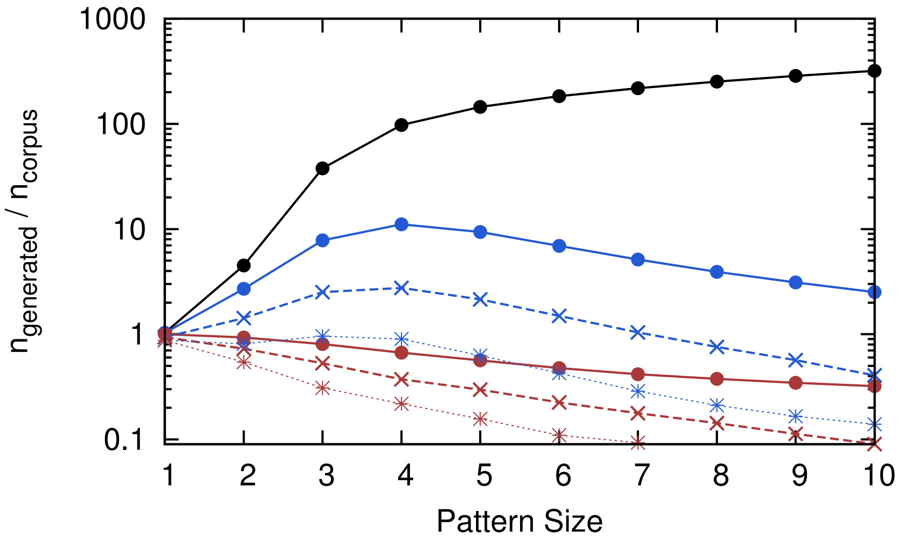

FIG. S3: **Style imitation and innovation: patterns at Hamming distance** $d = 0$ **and** $d = 1$ **from the corpus patterns.** For a given pattern size (x-axis), the plot shows $n^{(d)}_{\mathrm{generated}}/n_{\mathrm{corpus}}$ for $d = 0$ (red lines) and $d = 1$ (blue lines) as a function of pattern size. The generated sequences are of size $N = 500$ (dotted), $N = 5000$ (dashed) and $N = 50000$ (solid). As a reference we also counted the total number of patterns at $d = 1$ from patterns in the corpus (black line). For details on the corpus see Section S3

The figure can be read as follows. For a given size N of the generated sequence, the region bellow the red line shows the rate of imitation, the region between the red and blue lines shows the rate of innovation and the region between the blue and black lines shows the amount of patterns, at distance $d = 1$, that have been avoided by our model, because they failed to emerge from the competing pairwise constraints. This last point is important since

it shows that the model is highly selective when generating new patterns. We have listened to hundreds of patterns from the regions between the red and blue lines and found that the great majority of them where musically sensible alternatives to patterns from the corpus. On the other hand, patterns from the region between the blue and black lines tend to sound "wrong" since they often violate multiple pairwise constraints.

S5 Data-compression approach to measure cross-complexities and cross-entropies

In this section we report some details about the data-compression techniques we used to estimate the similarity between pairs of sequences. We followed in particular the approach proposed in [8, 9], based on the Lempel-Ziv algorithm [10]. We adopt in particular the notion of cross-complexity (from now onwards referred as cross-entropy) between two sequences of characters. Strictly speaking one refers to cross-entropy in information theory having in mind two sequences emitted from two specific sources. In Algorithmic Complexity Theory one deals with sequences without any reference to the sources that emitted them. In this case one speaks of cross-complexity between two sequences [11].

The cross-entropy between two sequences $\mathcal{A}$ and $\mathcal{B}$ is defined as the number of bits needed to encode each character emitted by the sequence $\mathcal{B}$ with the optimal coding for $\mathcal{A}$. Consider for instance two ergodic sources A and B emitting sequences of zeroes and ones: A emits 0 with probability p and 1 with probability $1-p$ whereas B emits 0 with probability q and 1 with probability $1-q$. The previously described compression algorithm can encode a sequence emitted by A almost optimal-coding a 0 with $-\log_2 p$ bits and a 1 with $-\log_2(1-p)$ bits. However, this A-optimal coding is not optimal for the sequence emitted by B. In fact, this

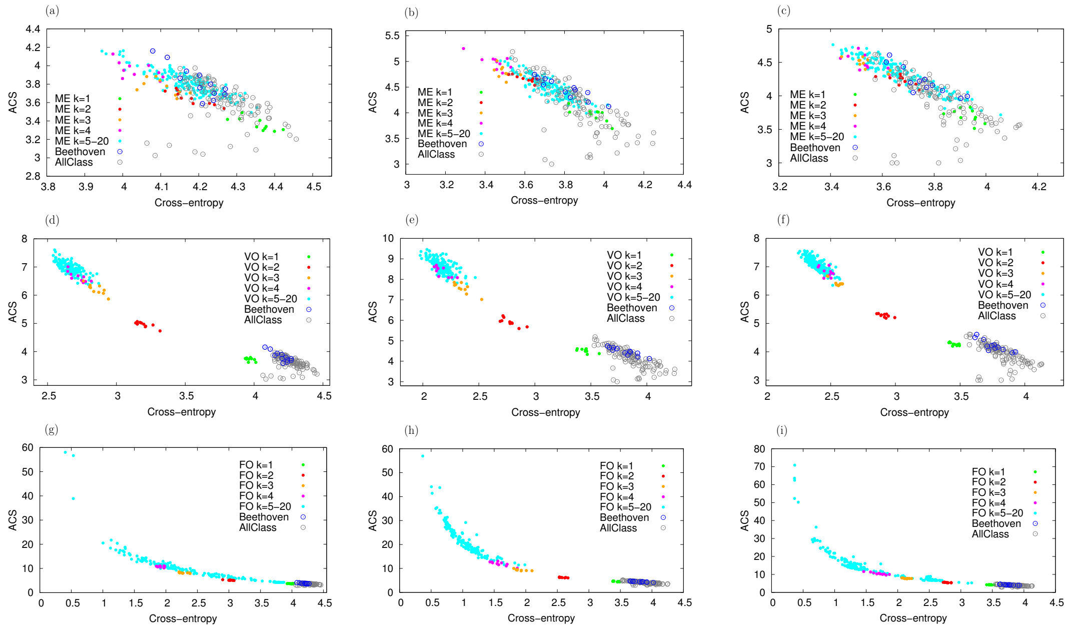

FIG. S4: **Borrowing vs. similarity in Beethoven.** This figure reports the Average Common Substring (ACS) vs. the values of the cross-entropies for all the artificial sequences generated with the Maximum Entropy (ME) model **(a), (b), (c)**, the variable-order (VO) Markov model **(d), (e), (f)** and the fixed-order (FO) Markov model **(g), (h), (i)**. Everything is computed with respect to three sequences by Ludwig van Beethoven (corresponding to the left, middle and right columns, see Section S3 for details). As in the main text, filled circles correspond to the artificial sequences. Colors code for the values of $K_{\max}$ in each different model. In addition in each panel the empty circles reports the same quantities for other original sequences of Beethoven (represented with blue circles) and other classical authors - Bach, Schumann, Chopin, Liszt and Albeniz - (AllClass represented with grey circles).

sequence's entropy per character in the A-optimal coding will be $-q\log_2 p-(1-q)\log_2(1-p)$. This is the cross-entropy between A and B. The entropy per character of the sequence that

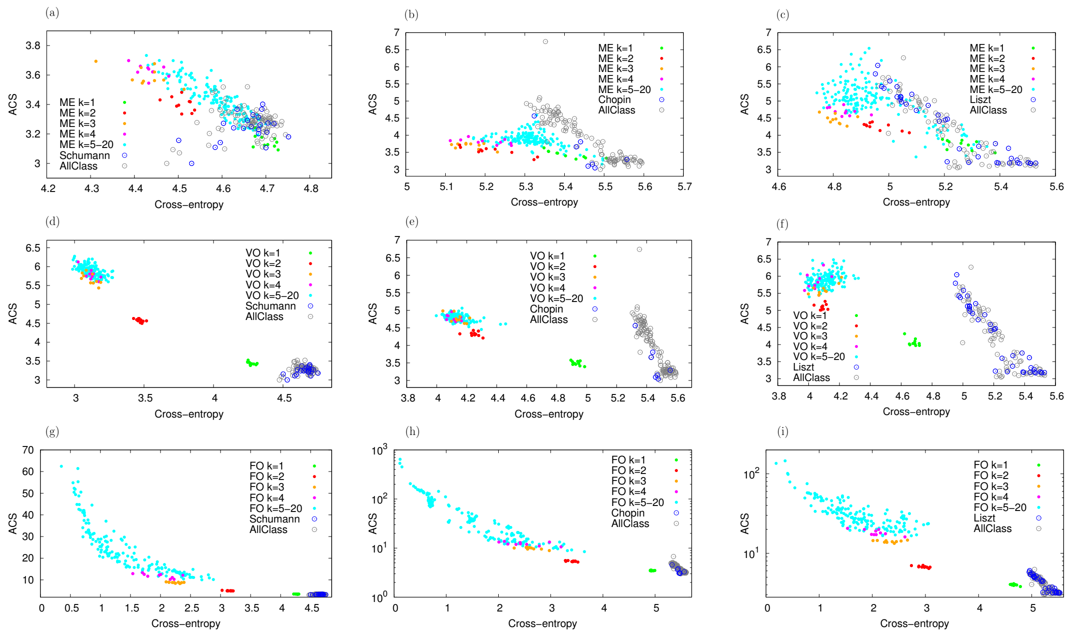

FIG. S5: **Borrowing vs. similarity in Schumann, Chopin and Liszt.** This figure reports the same results as in Fig. S4 for works by Robert Schumann **(a), (d), (g)**, Frédéric Chopin **(b), (e), (h)** and Franz Liszt **(c), (f), (i)**. For each composer AllClass means the set of all the composers (Bach, Beethoven, Schumann, Chopin, Liszt and Albeniz) excluding the composer considered. For details on the corpora used see Section S3.

B emits in its own optimal coding is $-q \log_2 q - (1-q)\log_2(1-q)$. The number of bits per character wasted to encode the sequence that B emits with the A-optimal coding is the relative entropy of A and B.

The approach proposed in [8, 9] estimates the cross-entropy between two sequences by using the LZ77 data compression scheme. The LZ77 algorithm first looks for duplicated strings in the input data. It replaces the second occurrence of a string with a pointer to the previous string. This pointer consists of two numbers: a distance, representing how far back into

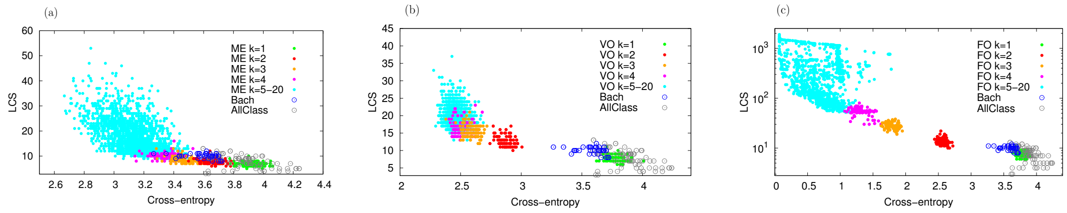

FIG. S6: **Borrowing vs. similarity.** This figure reports the Largest Common Substring (LCS) vs. the values of the cross-entropies for all the artificial sequences generated with the Maximum Entropy (ME) model **(a)**, the variable-order (VO) Markov model **(b)** and the fixed-order (FO) Markov model **(c)**. Everything is computed with respect to the sequence of J.S. Bach's first violin Partita (see Section S3 for details). Filled circles correspond to the artificial sequences. Colors code for the values of k in each different model. In addition in each panel the empty circles reports the same quantities for other original sequences of Bach (represented with blue circles) and other classical authors (AllClass represented with grey circles).

the window the sequence starts, and the length in characters of that subsequence. The original LZ77 algorithm defines the window as the section of the sequence already scanned sequentially. In our implementation of LZ77 we scan the sequence $\mathcal{B}$ by looking for matches only in sequence $\mathcal{A}$. In this way the algorithm is automatically looking for the best encoding of sequence $\mathcal{B}$ using the best code for sequence $\mathcal{A}$.

S6 Robustness of the results

In this section we report additional examples of the results presented in the section *Borrowing vs. Innovation* of the main text. We considers in particular more original corpora coming

from Beethoven, Schumann, Chopin and Liszt to demonstrate the robustness of the results. In particular, we show robustness both for different pieces of the same author (Fig. S4 presents the results for three different pieces by Ludwig Van Beethoven) and for pieces of different authors (Fig. S5 presents the results for pieces by Robert Schumann, Frédéric Chopin and Franz Liszt).

Finally we complement Figure 6 of the main text by reporting the same results where we substituted the Average Common Substring (ACS) with the Longest Common Substring (LCS).

S7 Audio files

We invite the reader to evaluate "musically" the output of our model by listening to the audio files we are providing. These where generated by models with $K_{\max} = 10$. The sequences belong to one of two categories, Classical and Jazz, depending on whether they where generated from sequences in the KdF or WJDB databases (see section S3). For every example we provide the original sequence, used as a training corpus, named `(PIECE NAME)_orig.mp3` and three artificial pieces, generated with the Maximum Entropy model, named `(PIECE NAME)_gen_(a, b or c).mp3`. The audio files are synthesized from MIDI information, which in turn is obtained by mapping the integer variable values output by our model to MIDI pitches.

Legend of the audio files:

`Bach_gen_a.wav` : SI audio 1 ;

`Bach_gen_b.wav` : SI audio 2 ;

Bach_gen_c.wav : SI audio 3 ;

Bach_orig_smaller.wav : SI audio 4 ;

Beethoven_gen_1_a.wav : SI audio 5 ;

Beethoven_gen_1_b.wav : SI audio 6 ;

Beethoven_gen_1_c.wav : SI audio 7 ;

Beethoven_gen_2_a.wav : SI audio 8 ;

Beethoven_gen_2_b.wav : SI audio 9 ;

Beethoven_gen_2_c.wav : SI audio 10 ;

Beethoven_gen_3_a.wav : SI audio 11 ;

Beethoven_gen_3_b.wav : SI audio 12 ;

Beethoven_gen_3_c.wav : SI audio 13 ;

Beethoven_orig_1_smaller.wav : SI audio 14 ;

Beethoven_orig_2_smaller.wav : SI audio 15 ;

Beethoven_orig_3_smaller.wav : SI audio 16 ;

Chopin_gen_a.wav : SI audio 17 ;

Chopin_gen_b.wav : SI audio 18 ;

Chopin_gen_c.wav : SI audio 19 ;

Chopin_orig_smaller.wav : SI audio 20 ;

Liszt_gen_a.wav : SI audio 21 ;

Liszt_gen_b.wav : SI audio 22 ;

Liszt_gen_c.wav : SI audio 23 ;

Liszt_orig_smaller.wav : SI audio 24 ;

Schumann_gen_a.wav : SI audio 25 ;

Schumann_gen_b.wav : SI audio 26 ;

Schumann_gen_c.wav : SI audio 27 ;

Schumann_orig_smaller.wav : SI audio 28 ;

CannonballAdderley_SoWhat_gen_a.wav : SI audio 29 ;

CannonballAdderley_SoWhat_gen_b.wav : SI audio 30 ;

CannonballAdderley_SoWhat_gen_c.wav : SI audio 31 ;

CannonballAdderley_SoWhat_orig.wav : SI audio 32 ;

CharlieParker_DonaLee_gen_a.wav : SI audio 33 ;

CharlieParker_DonaLee_gen_b.wav : SI audio 34 ;

CharlieParker_DonaLee_gen_c.wav : SI audio 35 ;

CharlieParker_DonaLee_orig.wav : SI audio 36 ;

DaveLiebman_SoftlyAsInAMorning_gen_a.wav : SI audio 37 ;

DaveLiebman_SoftlyAsInAMorning_gen_b.wav : SI audio 38 ;

DaveLiebman_SoftlyAsInAMorning_gen_c.wav : SI audio 39 ;

DaveLiebman_SoftlyAsInAMorning_orig.wav : SI audio 40 ;

JohnColtrane_GiantSteps_gen_a.wav : SI audio 41 ;

JohnColtrane_GiantSteps_gen_b.wav : SI audio 42 ;

`JohnColtrane_GiantSteps_gen_c.wav` : SI audio 43 ;

`JohnColtrane_GiantSteps_orig.wav` : SI audio 44 ;

`MilesDavis_Airegin_gen_a.wav` : SI audio 45 ;

`MilesDavis_Airegin_gen_b.wav` : SI audio 46 ;

`MilesDavis_Airegin_gen_c.wav` : SI audio 47 ;

`MilesDavis_Airegin_orig.wav` : SI audio 48 ;

---

[1] M. Weigt, R. a. White, H. Szurmant, J. a. Hoch, and T. Hwa, "Identification of direct residue contacts in protein-protein interaction by message passing", *Proceedings of the National Academy of Sciences of the United States of America*, vol. 106, pp. 67–72, (2009).

[2] R. Tibshirani, “Regression shrinkage and selection via the lasso,” *Journal of the Royal Statistical Society. Series B (Methodological)*, pp. 267–288, (1996).

[3] P. Ravikumar, M. J. Wainwright, J. D. Lafferty, *et al.*, "High-dimensional Ising model selection using ℓ_1-regularized logistic regression", *The Annals of Statistics*, vol. 38, no. 3, pp. 1287–1319, (2010).

[4] M. Schmidt, G. Fung, and R. Rosales, "Fast optimization methods for l1 regularization: A comparative study and two new approaches", in *Machine Learning: ECML 2007*, pp. 286–297, Springer, (2007).

[5] M. Schmidt, "Graphical model structure learning with l1-regularization". PhD thesis, University of British Columbia (Vancouver), (2010).

[6] WeimarJazzDatabase. http://jazzomat.hfm-weimar.de/dbformat/dboverview.html. The Weimar Jazz Database contains detailed transcriptions of famous jazz improvisations. As of March 2015 the database contains 257 songs.

[7] Kunst der Fuge Database. http://www.kunstderfuge.com/. Kunst der Fuge is a classical music MIDI database.

[8] D. Benedetto, E. Caglioti and V. Loreto, "Language Trees and Zipping", *Phys. Rev. Lett.* **88**, 048702 (2002).

[9] A. Baronchelli, D. Benedetto, E. Caglioti and V. Loreto, "Artificial sequences and complexity measures", *J. Stat. Mech.*, P04002 (2005).

[10] A. Lempel and J. Ziv, "A Universal Algorithm for Sequential Data Compression", *IEEE Trans. Inf. Theory* **23**, 337–343 (1977).

[11] D. Cerra and M. Datcu, "Algorithmic Relative Complexity ", *Entropy* **13**, 902–914 (2011).
